# Decitabine and SAHA-Induced Apoptosis Is Accompanied by Survivin Downregulation and Potentiated by ATRA in p53-Deficient Cells

**DOI:** 10.1155/2014/165303

**Published:** 2014-07-21

**Authors:** Barbora Brodská, Petra Otevřelová, Aleš Holoubek

**Affiliations:** Institute of Hematology and Blood Transfusion, U Nemocnice 1, 128 20 Prague 2, Czech Republic

## Abstract

While p53-dependent apoptosis is triggered by combination of methyltransferase inhibitor decitabine (DAC) and histone deacetylase inhibitor suberoylanilide hydroxamic acid (SAHA) in leukemic cell line CML-T1, reactive oxygen species (ROS) generation as well as survivin and Bcl-2 deregulation participated in DAC + SAHA-induced apoptosis in p53-deficient HL-60 cell line. Moreover, decrease of survivin expression level is accompanied by its delocalization from centromere-related position in mitotic cells suggesting that both antiapoptotic and cell cycle regulation roles of survivin are affected by DAC + SAHA action. Addition of subtoxic concentration of all-trans-retinoic acid (ATRA) increases the efficiency of DAC + SAHA combination on viability, apoptosis induction, and ROS generation in HL-60 cells but has no effect in CML-T1 cell line. Peripheral blood lymphocytes from healthy donors showed no damage induced by DAC + SAHA + ATRA combination. Therefore, combination of ATRA with DAC and SAHA represents promising tool for therapy of leukemic disease with nonfunctional p53 signalization.

## 1. Introduction

Apoptosis is an essential process ensuring correct organism development by the cell elimination when the role of cell was finished or when irreparable cellular damage occurred. Failure of apoptotic mechanism leads to abnormal cell proliferation or differentiation arrest giving rise to tumor formation and cancer development. Therefore, mechanisms of apoptotic pathways are widely investigated and the causes of incorrect apoptotic process are clarified. Various anticancer drugs are tested for their ability to reestablish proper apoptotic machinery in cancer cells with minimal effect on surrounding tissue or normal cells.

Leukemia belongs to a large group of diseases in which variability of factors contributing to tumor development requires individual therapeutic approach. Growing number of prognostic factors helps to better define optimal therapy regimen, but extensive adverse effects together with acquired resistance to chemotherapy are calling for novel therapy strategies in leukemia. Combination of two or more drugs often targets more specifically the tumor cells while sparing the normal cells and, thus, reduces therapy-related side effects.

The epigenetic drug decitabine (Dacogen, DAC) is a cytosine analog causing DNA methyltransferase (DNMT) inhibition. It has been shown to be efficient in numerous clinical studies including intermediate-1 to high risk patients with secondary acute myeloid leukemia (AML) arising from myelodysplastic syndrome (MDS) and in MDS with poor-risk cytogenetics [1, 2]. Beside its single-agent efficiency, it has been studied in various combination treatment regimens, especially in combination with other epigenetic drugs. In hematological malignancies, combination regimens with histone deacetylase (HDAC) inhibitors are clinically tested (http://www.clinicaltrial.gov/).

HDAC inhibitors cause increased acetylation of histones as well as of nonhistone proteins leading to protein conformational changes and subsequent changes of their activity. Reactivation of epigenetically silenced tumor suppressors or repression of overexpressed prosurvival genes by combined action of DNMT and HDAC inhibitors was detected in many cancer types [3–6].

In our previous work [5] we found that 1 *μ*M decitabine induces p53-dependent apoptosis and that this phenomenon is augmented by 1 *μ*M SAHA addition. p53-deficient cells also showed signs of apoptosis with ROS generation as the most pronounced phenomena. Slight attenuation of Bcl-2 mRNA and protein expression was also detected, but none of these effects could successfully elucidate the extent of apoptosis observed in p53-deficient cells after DAC + SAHA treatment. Therefore, searching for other possible mechanisms contributing to DAC + SAHA-induced apoptosis in p53-deficient cells is needed. Among the apoptosis related proteins, survivin seems to be promising candidate to have a role in apoptosis triggered by DAC + SAHA in p53-deficient cells. Survivin belongs into inhibitors-of-apoptosis (IAP) family and operates as antagonist of apoptotic process. Survivin is frequently overexpressed in tumors [7, 8] while its presence in normal cells is rare. Besides its function as inhibitor of apoptosis, survivin has a role in regulation of mitosis as important component of chromosomal passenger complex (CPC). Chromosomal passenger complex responsible for microtubule association to kinetochores consists of Aurora B, INCENP, Borealin, and Survivin, each of them being necessary for CPC function [9, 10]. In this complex, survivin mediates docking of Aurora B kinase ensuring the attachment of the mitotic spindle to the centromere [11]. Expression of survivin is, therefore, enhanced in G2/M phase of cell cycle [9]. Deregulation of survivin prolongs metaphase and induces polyploidy in HeLa cells [12], survivin overexpression may obliterate G2/M checkpoint and allow aberrant progression of cells through mitosis [13]. Combined treatment of SCLC cells with decitabine and histone deacetylation inhibitor valproic acid (VPA) led to significant reduction of survivin protein [14]. Decitabine combined with trichostatin A (TSA) downregulated survivin mRNA in SKM-1 cells [15]. Therefore, we investigated the role of survivin in DAC + SAHA-induced apoptosis in p53 deficient cell line HL-60.

There is much evidence that expression of survivin correlates with Bcl-2 expression [16, 17] and that Bcl-2 overexpression abolished SAHA-induced apoptosis without affecting its differentiation or cell cycle regulatory effects [18–20]. On the other hand, effect of SAHA is possible to increase by adding all-trans retinoic acid (ATRA, [21]), which is known to suppress Bcl-2 expression [22–25]. Deregulation of Bcl2 accompanies all-trans retinoic acid- (ATRA-) induced granulocytic differentiation and subsequent apoptosis in HL-60 cell line [26]. Concurrently, 96 h ATRA exposure reduces survivin levels in HL-60 cells [27]. Moreover, DAC and/or SAHA combined with ATRA induced apoptosis in ATRA-resistant NB-4 cells [28]. Effect of combined DAC + SAHA + ATRA treatment was investigated in second part of this paper. Retinoic acid is known to induce differentiation and apoptosis in different tumor cell types. ATRA causes sensitization to TRAIL-induced apoptosis in NSCLC-derived cell lines [29] or induces apoptosis in prostate cancer cells [30], complete remission occurs in the majority of patients with acute promyelocytic leukemia (APL) after ATRA treatment [31]. In APL-derived cell lines, ATRA induces differentiation followed by apoptosis [32, 33]. ATRA has only limited single agent activity in AML without the PML-RAR*α* fusion (non-M3 AML). However, pretreatment with decitabine or entinostat primed AML cell lines Kasumi-1 and HL-60 to ATRA-induced differentiation [34].

## 2. Material and Methods

### 2.1. Cell Culture and Chemicals

Peripheral blood lymphocytes of healthy donors were isolated from buffy coats on Histopaque 1077 (Sigma-Aldrich) as described earlier [35]. Leukemia cell line CML-T1 (German Collection of Microorganisms and Cell Cultures, Braunschweig, Germany) and HL-60 (European Collection of Animal Cell Cultures, Great Britain) were cultivated in RPMI 1640 (Biochrom AG, Germany) supplemented with 10% FCS, 37°C, and 5% CO_2_ atmosphere. Decitabine, ATRA (both from Sigma-Aldrich), and SAHA (Cayman Chemical Company, Ann Arbor, MI, USA) were added from 1–100 mM stock solution to final concentration of 1 *μ*M for 48 h. N-acetyl-L-cysteine (NAC, Sigma-Aldrich) and caspase inhibitors z-VAD-fmk (Santa Cruz) and Q-VD-OPh (R&D Systems) were added to final concentration of 10 mM (NAC), 50 *μ*M (z-VAD-fmk), and 10–30 *μ*M (Q-VD-OPh), respectively.

### 2.2. Viability Test

The viability (metabolic activity) of cells preincubated in microplates with DAC, SAHA, ATRA, and their combinations for 48 h was monitored using an MTT Kit I (Roche Diagnostics Corporation, Indianapolis, IN, USA). Following the procedure described earlier [35], the absorbance of reporter substrate was measured on an ELISA reader (MTX Lab Systems, Inc. Vienna, VA, USA).

### 2.3. Flow Cytometry

Distribution of cell cycle phases (monitored by propidium iodide, PI), generation of reactive oxygen species (ROS, observed by dichlorodihydrofluorescein diacetate, H_2_DCFDA), the extent of apoptosis (defined by Annexin V-FITC positive/PI negative population of treated cells) and apoptotic proteins survivin, and BAX expression together with Hoechst-stained DNA content were investigated by flow cytometry. All fluorescent probes and Alexa Dyes-conjugated secondary antibodies were purchased from Life Technologies Corporation, rabbit anti-survivin and mouse anti-BAX primary antibodies were from Santa Cruz Biotechnology. All samples (50 000 events/sample) were analyzed on an LSRFortessa cell analyzer (BD Biosciences).

*Cell Cycle*. Cells treated by DAC + SAHA and/or ATRA for 48 h were harvested, washed twice with 2 mL of PBS, resuspended in 500 *μ*L of PBS, added into 4.5 mL of ice-cold 70% EtOH, and stored at −20°C. For the analysis, suspension was extensively washed with PBS, resuspended in PI-staining solution (0.1% (v/v) Triton X-100; 100 *μ*g/mL RNaseA, 50 *μ*g/mL PI), and kept for 30 min in the dark.

*ROS Production*. Treated cells were harvested, washed with PBS, and suspended in 1 mL PBS. H_2_DCFDA was added to final concentration of 10 *μ*M for 30 min in 5% CO_2_ at 37°C.

*Apoptosis*. Cells were harvested, washed with PBS, and resuspended in 100 *μ*L of Annexin Binding Buffer (ABB: 10 mM Hepes pH 7.4, 140 mM NaCl and 2.5 mM CaCl_2_) containing 5 *μ*L of Annexin V-FITC solution. After 15 min in the dark (RT), 400 *μ*L of ABB was added and suspension was incubated for another 5 min with 2 *μ*L of 250 *μ*g/mL PI stock solution.

*Immunostaining*. Treated cells were harvested, washed with PBS, and suspended in 200 *μ*L of 4% paraformaldehyde (PFA) for 15 min at RT. After 10 min/500 g/RT, centrifugation cells were suspended at 50 *μ*L of ice-cold PBS and 450 *μ*L of ice-cold MetOH was added. Samples were stored at −20°C at least overnight. For immunostaining, the cells were blocked with 1% BSA (in PBS) for 20 min, incubated for 1 h with mouse monoclonal anti-Bax and rabbit polyclonal anti-survivin (both from Santa Cruz Biotechnology) primary antibodies (1 : 100) and for 30 min with the mixture of secondary antibodies (AlexaFluor488-conjugated anti-rabbit and AlexaFluor647-conjugated anti-mouse, 1 : 200) with 1 *μ*M Hoechst33342.

### 2.4. RNA Isolation and qRT-PCR

RNA of total 5 × 10^6^ cells was isolated with RNeasy kit (Qiagen) according to manufacturers' instructions and deposited in −80°C until use. mRNA quality and concentration were assessed on ND-1000 Nanodrop system and qRT-PCR was performed on CFX96 real-time system (BioRad) using SensiFAST SYBR No-ROX One-Step Kit (Bioline). Relative mRNA quantity and standard deviations were calculated with CFX Manager Software. *β*-actin was used as reference gene. Primers for RT-PCR were designed with PrimerBLAST software (NCBI).

### 2.5. Immunoblotting

For immunoblotting, cells were lysed in Lytic Buffer (LB: 50 mM Tris.HCl pH 7.5, 1% NP-40, 5 mM EDTA, 150 mM NaCl, 1 mM DTT, 1 mM PMSF, 4 *μ*L/1 mL protease inhibitor cocktail and 5 *μ*L/1 mL phosphatase inhibitor cocktail 2, both cocktails were from Sigma-Aldrich) for 30 min at 4°C and then centrifuged at 10.000 g/30 min/4°C. After protein concentration assay (Bradford), supernatants were mixed with 2xLaemmli sample buffer and boiled for 5 min. Five micrograms of total protein were then subjected to SDS-PAGE and transferred into nitrocellulose membrane (Hybond PVDF, Amersham). Rabbit polyclonal primary antibodies against Bcl-2, p21, PARP, Puma, and survivin as well as mouse monoclonal anti-p53 and anti-BAX were from Santa Cruz Biotechnology. Rabbit monoclonal anti-caspase-3 (both procaspase and active form) originated from Abcam. Mouse monoclonal anti-*β*-Actin (Santa Cruz Biotechnology) was used to detect *β*-Actin expression as control of equal loading. All primary antibodies except anti-PARP (1 : 1,000) and anti-caspase-3 (1 : 5,000) were used at dilution 1 : 500. Anti-rabbit and anti-mouse HRP-conjugated secondary antibodies were purchased from Thermo Scientific and used in concentrations 1 : 50,000–1 : 100,000. ECL Plus Western Blotting Detection System (Amersham) was used for chemiluminescence visualization and evaluation by G-box iChemi XT4 digital imaging device (Syngene Europe, Cambridge). Relative protein expression was evaluated by GeneTools Software and statistical analysis was performed in GraphPad software.

### 2.6. Fluorescence Microscopy

Cells in suspension were seeded on coverslip in humidified chamber for 15 min and then fixed with 4% paraformaldehyde (PFA) overnight. After 10 min of permeabilization by 0.5% Triton X-100, the cells were incubated for 1 h with mouse monoclonal anti-Bax and rabbit polyclonal anti-survivin (both from Santa Cruz Biotechnology) or rabbit monoclonal anti-COX IV (Cell Signalling Technology) primary antibodies (1 : 100) and for another 1 h with the mixture of secondary antibodies (AlexaFluor488-conjugated anti-rabbit and AlexaFluor647-conjugated anti-mouse, both from LifeTechnologies, 1 : 200) with Hoechst33342 (1 *μ*M, LifeTechnologies). After extensive washing by PBS-Tween, stained cells were mounted into Prolong mounting suspension and observed under confocal laser scanning microscope FluoView FV1000 (Olympus Corporation). Fluorescence images were processed by Fluoview software FV10-ASW 3.1.

### 2.7. Statistical Analysis

In diagrams, arithmetic means of at least three times repeated experiments were plotted with SEM error bars. Significance levels (*P* values of *t*-tests) were determined using InStat Software (GraphPad Software). A *P* value of 0.05 or lower was considered to be indicative of a statistically significant difference between groups compared.

## 3. Results

### 3.1. Survivin in DAC + SAHA-Treatment

**Figure 1:**
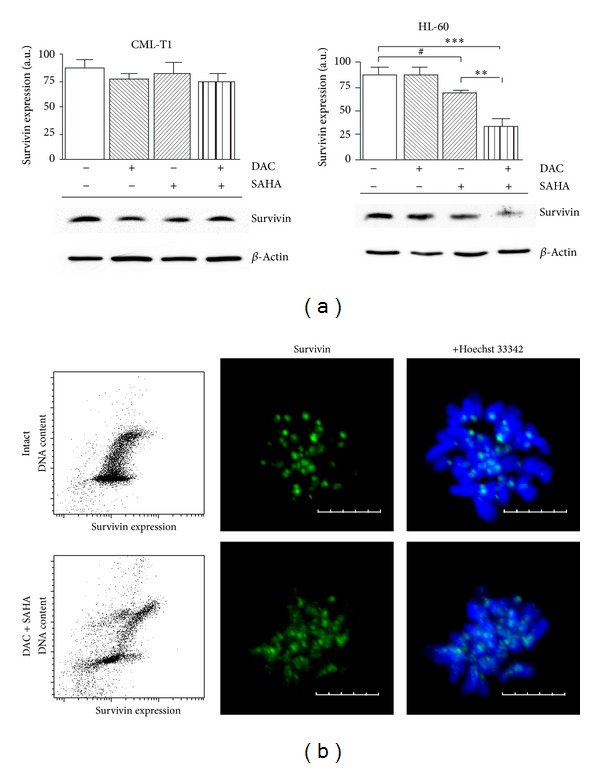
(a) Effect of 1 *μ*M DAC and/or 1 *μ*M SAHA on survivin expression after 48 hours treatment. Error bars represent ±SEM and immunoblots from cell lysates of leukemic cell lines CML-T1 and HL-60 are representatives of at least five experiments with similar results. Statistical significance degree of difference between treated samples and the corresponding control: *P* = 0.053 (#), *P* < 0.01 (∗∗), *P* < 0.001 (∗∗∗). (b) Untreated or DAC + SAHA-treated cells of HL-60 cell line were permeabilized and stained for survivin (Alexa Fluor 488, green) and DNA content (Hoechst 33342, blue). Fluorescence intensity distribution and intracellular localization were assessed by flow-cytometry and confocal microscopy. Scale bar represents 5 *μ*m.

In our previous work [5] we documented the efficacy of histone deacetylase inhibitor SAHA in combination with decitabine on leukemia-derived cell lines with respect to their p53 status. While p53-dependent pathway was proved to be accountable to apoptotic cell death in p53wt cells, reactive oxygen species (ROS) generation affected apoptosis in p53-deficient cells. On the basis of literature data, we monitored changes in survivin expression during DAC and SAHA treatment of leukemic cell lines. While in p53wt cell line (CML-T1) no substantial changes were observed, considerable decrease of survivin expression was detected in p53-deficient HL-60 cell line treated by DAC + SAHA (Figure 1(a)). Moderate survivin attenuation was observed also in HL-60 cells treated with SAHA alone, but this fact had no effect on cell viability and apoptosis in 48 h time frame.

Decitabine, alone or in combination with SAHA, causes increase in cell fraction in G2/M phase in HL-60 cells [5]. Therefore, G2/M phase-related accumulation of survivin could be expected in DAC + SAHA-treated cells instead of attenuation of protein expression. Reduction of survivin protein expression, thus, could not be explained by DAC + SAHA-induced cell cycle distribution changes. Flow-cytometric assessment of anti-survivin labeled cells showed decrease of fluorescence in all cell cycle phases (Figure 1(b)) after DAC + SAHA treatment. Separate populations with low survivin expression correspond to region indicating damaged cells in FSC/SSC plot. Confocal microscopy visualization of immunofluorescently labeled PFA-fixed cells revealed partial delocalization of centromere-related survivin induced by DAC + SAHA combination (Figure 1(b)). Taken together, downregulation of survivin expression irrespective of cell cycle phase and its aberrant localization out of centromeres during mitotic phase are associated with apoptosis in DAC + SAHA treated p53-deficient cells. Interestingly, broadening of survivin fluorescence intensity distribution was detectable in DAC + SAHA-treated CML-T1 cells, irrespective of the absence of protein level change in immunoblot-visualized overall expression. However, localization of centromere-related survivin remained unchanged in this cell line.

### 3.2. Effect of All-Trans Retinoic Acid

**Figure 2:**
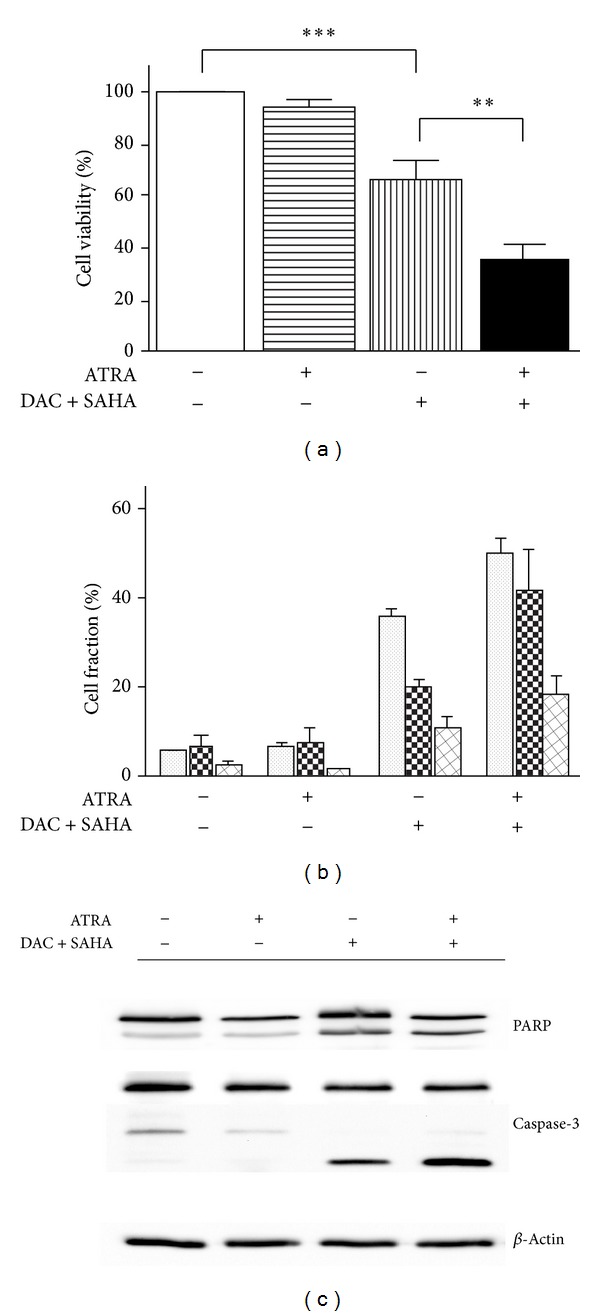
Apoptosis induction by ATRA and/or DAC + SAHA treatment: HL-60 cells were treated for 48 h by 1 *μ*M ATRA and/or DAC + SAHA combination. (a) Cell viability assessed by MTT test. Error bars represent ±SEM of at least 5 measurements. Statistical significance degree of difference between treated samples and the corresponding control: *P* < 0.05 (∗), *P* < 0.01 (∗∗), *P* < 0.001 (∗∗∗). (b) Fraction of cells in subG1 phase (dotted columns); PARP fragmentation: f89/f116 ratio (checked columns); Annexin V+/PI-fraction of Annexin V-FITC/PI stained cells (hatched columns). Error bars represent ±SEM of at least 3 measurements. (c) Immunoblots showing PARP fragmentation and caspase-3 activation after 48 h ATRA and/or DAC + SAHA treatment in HL-60 cells and *β*-actin expression as a loading control. Images are representatives of at least three experiments.

**Figure 3:**
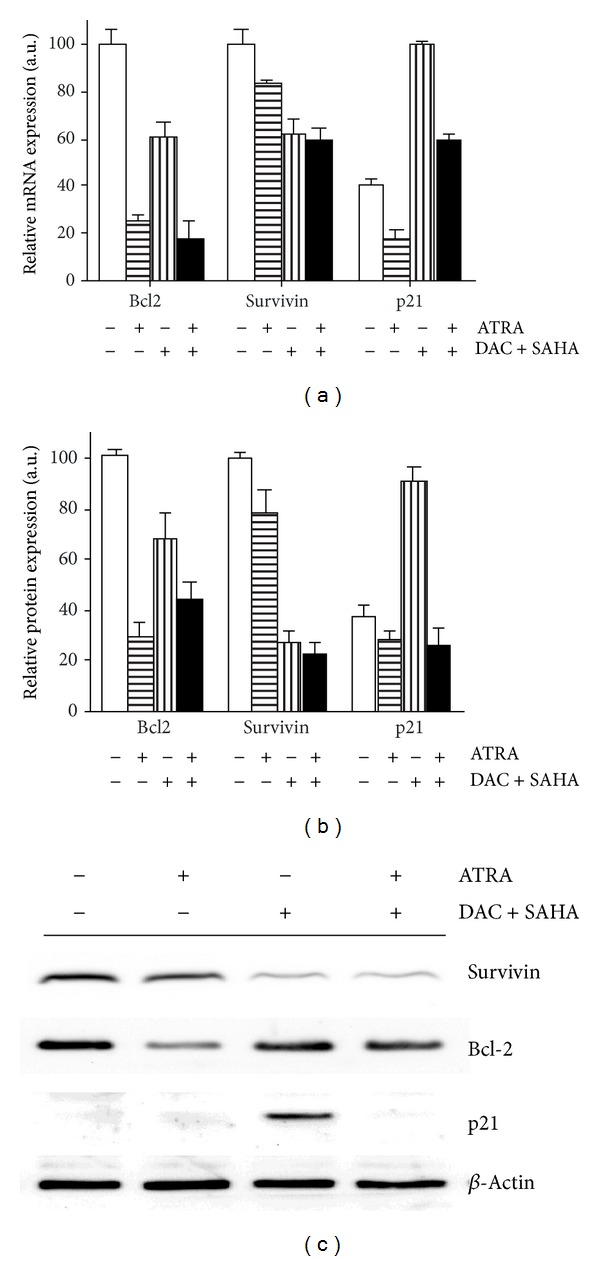
Expression of Bcl-2, survivin, and p21WAF1 in HL-60 cells after 48 h of ATRA and/or DAC + SAHA treatment. (a) Transcription levels monitored by qRT-PCR. (b) Protein expression monitored by immunoblotting. Error bars represent ±SEM of three measurements. (c) Representative immunoblots with *β*-actin expression as a loading control.

Moderate attenuation of Bcl-2 expression was observed after DAC + SAHA treatment of HL-60 cells [5]. Large Bcl-2 reduction was widely documented in HL-60 cells as a consequence of all-trans retinoic acid (ATRA) treatment [16, 26]. Therefore, we tested the effect of therapeutic concentration (1 *μ*M) of ATRA alone or in combination with DAC + SAHA on apoptotic features and apoptosis-related protein expression. Cell viability and overall apoptotic features (Annexin V staining, PARP fragmentation, executive caspases activation, subG1 cell fraction) remained unchanged during 48 h of ATRA treatment, while the presence of ATRA together with DAC + SAHA substantially augmented both the viability drop and apoptosis in comparison with cells treated by DAC + SAHA (Figure 2). Viability of HL-60 cells dropped to 36% in presence of DAC + SAHA + ATRA in comparison with 66% observed after DAC + SAHA treatment and fraction of cells in subG1 phase increased from 35% to 50%. None of DAC + ATRA and SAHA + ATRA combinations caused apoptosis comparable even to DAC + SAHA effect. Concurrent addition of all three drugs was the most efficient way of apoptosis triggering by these drugs combination. Addition of ATRA 24 h before or after DAC + SAHA combination brought similar effect, but one-shot regimen manifested the most statistically significant results (data not shown). Persisting survivin dowregulation and Bcl-2 attenuation were detected at both mRNA and protein levels in comparison with cells treated with DAC + SAHA (Figure 3). SAHA-induced upregulation of cyclin-dependent kinase inhibitor p21WAF1 was observed in DAC + SAHA-treated cells [5]. However, this upregulation diminished with ATRA addition and treatment with ATRA alone even slightly decreased p21 level in HL-60 cells (Figure 3).

**Figure 4:**
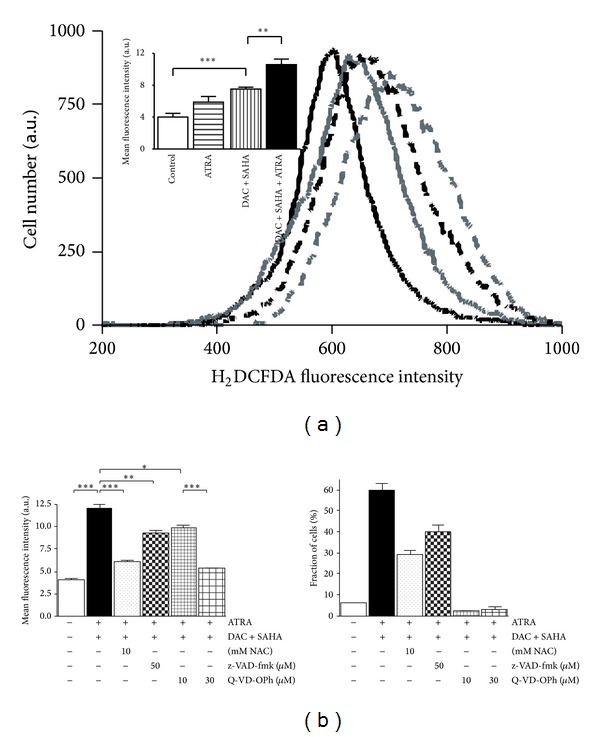
(a) Production of reactive oxygen species by control cells (black, full line) and HL-60 cells treated for 48 h by 1 *μ*M ATRA (grey, full line), DAC + SAHA (black, dashed line), or DAC + SAHA + ATRA combination (grey, dashed line) monitored by flow-cytometric analysis of H_2_DCFDA staining. Embedded chart: mean value of flow-cytometer-detected fluorescence intensity was analyzed. (b) NAC for reduction of ROS generation or caspases inhibitors z-VAD-fmk or Q-VD-OPh were used in concentrations as indicated and ROS generation as well as fraction of cells in subG1 phase were measured by flow cytometry. Error bars represent ±SEM of three measurements. Statistical significance degree between corresponding samples was analyzed and *P* value evaluated as *P* < 0.05 (∗), *P* < 0.01 (∗∗), *P* < 0.001 (∗∗∗).

Reactive oxygen species (ROS) induction was reported to play a role in DAC + SAHA-induced apoptosis of HL-60 cells [5]. Significant amplification of this effect was observed after ATRA addition, when slight increase induced by ATRA alone substantially potentiated ROS generation induced by DAC + SAHA (Figure 4(a)). Relationship between ROS generation and the extent of apoptosis was further investigated using ROS-scavenger N-acetyl-cysteine (NAC) and two pan-caspase inhibitors, z-VAD-fmk or Q-VD-OPh. Significant attenuation of viability drop, Annexin positivity, and subG1 cell fraction together with inhibition of ROS generation has been observed with 10 mM NAC added to DAC + SAHA + ATRA-treated cells. Similarly, both 50 *μ*M z-VAD-fmk and 10 *μ*M Q-VD-OPh caused significant reduction of apoptotic features as well as attenuation of ROS generation. When added at 30 *μ*M concentration, Q-VD-OPh completely cancelled apoptotic indicators and further reduced ROS generation (Figure 4(b)).

**Figure 5:**
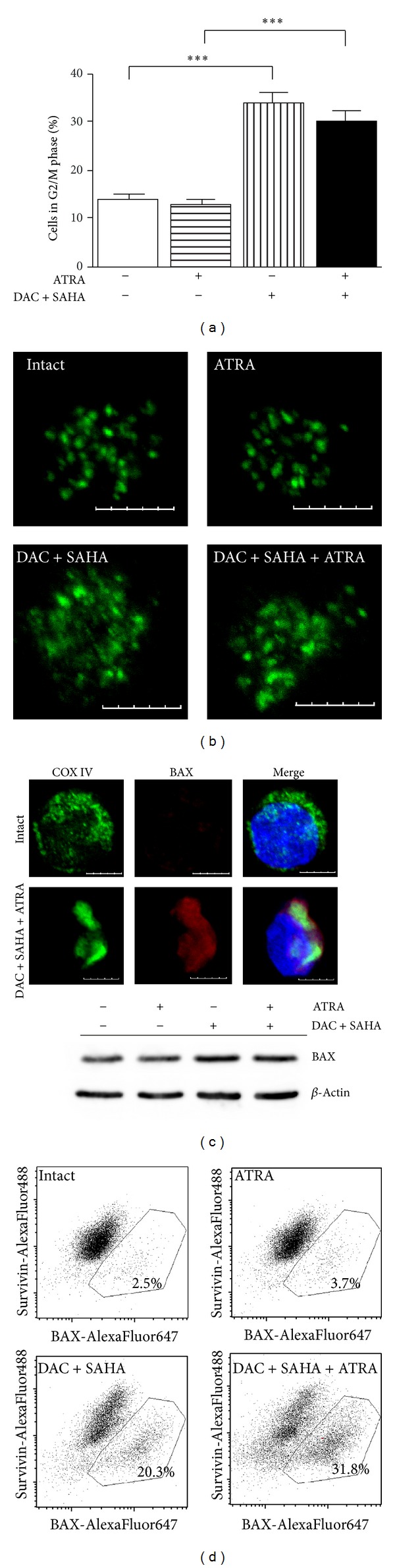
(a) Fraction of non-subG1 cells in G2/M cell cycle phase monitored by PI-staining of ethanol-fixed cells treated by 1 *μ*M ATRA, DAC + SAHA, or DAC + SAHA + ATRA combination. Data are averaged from at least three experiments. Statistical significance degree between corresponding samples was analyzed and *P* value evaluated as *P* < 0.05 (∗), *P* < 0.01 (∗∗), *P* < 0.001 (∗∗∗). (b) Analysis of survivin (AlexaFluor488, green) and (c) BAX (AlexaFluor647, red) expression and localization in DAC + SAHA and/or ATRA-treated HL-60 cell line by confocal microscopy and (d) flow cytometry. In (c), mitochondria are visualised by COX IV (AlexaFluor488, green) and nuclear DNA stained by Hoechst 33342 (blue). Scale bar in microscopy images represents 5 *μ*m.

Effect of enhanced cell fraction in G2/M phase of DAC + SAHA-treated cells partially persists even in cells treated with DAC + SAHA + ATRA combination. From these cells, more than 14% (30% of viable cells) displayed 2n-DNA content in cell cycle distribution, but only a few of them had condensed chromatin characteristic for mitotic cells. In these rarely appearing mitotic cells, survivin was only partially delocalized from centromere in comparison with its large delocalization observed for DAC + SAHA (Figures 5(a) and 5(b)). We conclude that ATRA accelerates disintegration and apoptosis of cells with DAC + SAHA-induced G2/M arrest and with delocalized survivin.

Despite the fact that no significant changes were detected in overall expression of proapoptotic Bcl-2-family protein BAX, its relocalization into mitochondria indicating mitochondrial apoptotic pathway triggering was observed in apoptotic DAC + SAHA-treated cells [5]. Mitochondrial localization of active BAX was detected also after DAC + SAHA + ATRA treatment although BAX protein expression from whole cell lysate only slightly increased (Figure 5(c)). Flow-cytometric assessment of immunofluorescently labelled fixed cells revealed negative relation between survivin and BAX expression: population of cells with low survivin expression showed higher intensity of BAX staining (Figure 5(d)).

**Figure 6:**
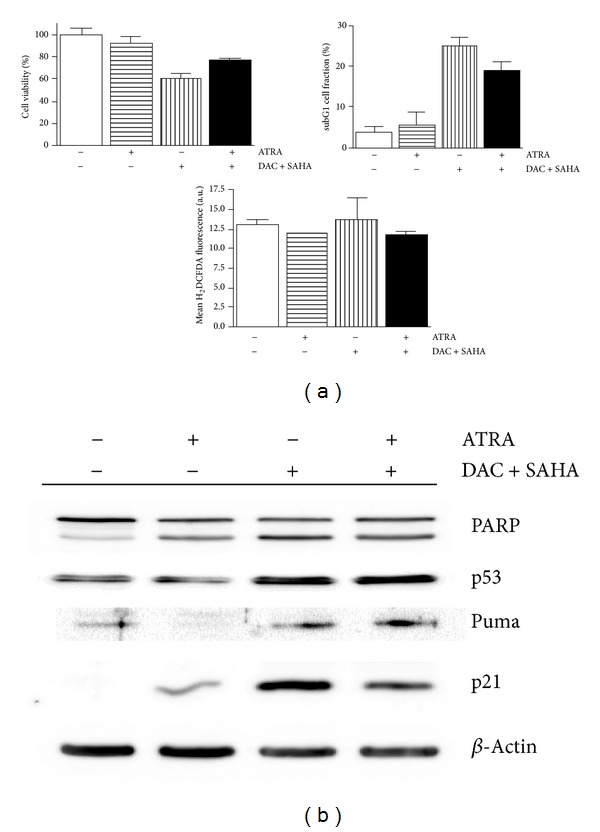
(a) Viability, subG1 cell fraction, and ROS production in control cells and after 48 h ATRA, DAC + SAHA or DAC + SAHA + ATRA treatment of CML-T1 leukemic cell line. Error bars represent ±SEM of at least three measurements. (b) Fragmentation of PARP, expression of tumor suppressor p53, and proteins related to apoptosis monitored in CML-T1 cells by immunoblotting. Images are representatives of three experiments with similar results.

**Figure 7:**
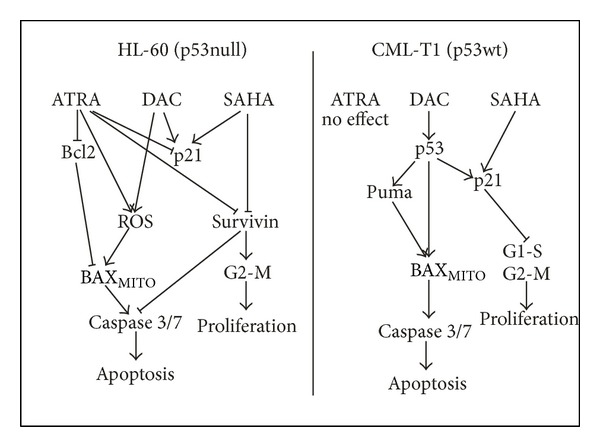
Schematic representation of the proposed pathway of DAC + SAHA + ATRA action with respect to p53 status.

ATRA addition to DAC + SAHA-treated cells of p53wt-possessing leukemia cell line CML-T1 did not bring any amplification of effect on viability, apoptosis, and ROS production (Figure 6(a)). p53-induced apoptotic pathway with Puma and p21WAF1 upregulation seems to stay to be the main mechanism of apoptosis induced by DAC + SAHA even after ATRA addition (Figure 6(b)).  Figure 7 illustrates schematic representation of the proposed pathway of DAC + SAHA + ATRA action in both HL-60 (p53null) and CML-T1 (p53wt).

In our previous work [5], we found that even 72 h DAC + SAHA treatment of peripheral blood lymphocytes of healthy donor (PBL) displayed no significant effect on their viability and apoptosis. Therefore, we tested effect of ATRA addition to intact or DAC + SAHA-treated PBL. ATRA addition did not bring any change to PBL resistance observed in experiments with DAC + SAHA (data not shown).

## 4. Discussion

Combination chemotherapy is frequently used in cancer treatment to prevent drug resistance development. Another goal of the combination therapy is minimization of intolerable side effects. Optimal combinations, doses, and regimens for various cancer types are investigated in many clinical trials. Epigenetic drugs targeting methylation and acetylation processes have been shown effective in various combinations. Recently, we reported that combination of DNA-methyltransferase inhibitor decitabine (DAC) and histone deacetylase inhibitor SAHA triggered p53-dependent apoptosis in leukemia-derived cell line CML-T1. However, apoptotic features were observed also in p53-defficient leukemia cell line HL-60. Generation of reactive oxygen species was suggested to play a role in this apoptotic process. Beside a high number of cells in subG1 phase, substantial cell fraction was in G2/M phase and microscopic observation revealed many aberrantly large cells with multiple nuclei in pool of DAC + SAHA-treated cells. We, therefore, decided to investigate the role of survivin, a protein with a dual role in both apoptosis and mitotic spindle checkpoint. Survivin promoter contains approximately 250ntd region of canonic CpG islands, but this region is unmethylated in the majority of both normal and neoplastic tissues [9]. Exception from this fact was observed only in endometrial tumor cells, which had hypermethylated survivin promoter and were sensitized by decitabine to p53-dependent survivin repression [36]. Therefore, DNA-methyltransferase inhibitor should not affect survivin promoter activity in HL-60 cells. Accordingly, we did not detect changes of survivin expression in decitabine-treated cells. On the other hand, SAHA was referred to decrease survivin level, when it is used in higher than 1 *μ*M concentration and/or for longer time (3–7 days) [37]. Moreover, various histone deacetylase inhibitors in combination with DNA-methyltransferase inhibitor decitabine downregulated survivin in both mRNA and protein levels [14, 15]. In our experiments, slight survivin downregulation was observed after 48 h treatment of HL-60 cells with 1 *μ*M SAHA, but this decrease was not statistically significant (*P* = 0.053). However, in cells treated by DAC + SAHA combination, considerable attenuation of survivin expression was detected by immunoblot (Figure 1(a)). Downregulation of survivin level was observed in all cell cycle phases and immunofluorescence staining investigated by confocal microscopy manifested delocalization of survivin from its centromere-related position in mitotic cells (Figure 1(b)). In summary, both the antiapoptotic and cell-cycle regulating roles of survivin were affected by DAC + SAHA treatment indicating that certain degree of connectivity should exist between these two roles of survivin in p53-deficient cells.

Relation between expression of survivin and another antiapoptotic protein, Bcl-2, was found in different tumour tissues. Positive correlation was found in nonsmall cell lung carcinoma (NSCLC), where high expression of both proteins negatively correlated with postoperative survival duration [16]. In hepatocellular carcinoma cells (HCC), combination of survivin and Bcl-2 inhibitors led to large apoptotic effect [17]. Survivin together with Bcl-2 was downregulated by yttrium-90 treatment of chemorefractory liver-dominant metastatic colorectal cancer [38]. Moderate Bcl-2 decrease was observed in both mRNA and protein levels after DAC + SAHA treatment of HL-60 cell line [5]. Therefore, effect of all-trans retinoic acid (ATRA), which is known to deregulate Bcl-2 in many cancer cell types [22–25], was investigated in intact or DAC + SAHA treated cells. 1 *μ*M ATRA alone caused extensive diminution of Bcl-2 expression without significant changes of viability and apoptotic features. Conversely, ATRA addition to DAC + SAHA caused only slight Bcl-2 downregulation, but large viability decrease as well as increasing values of apoptotic features was observed in ATRA + DAC + SAHA-treated cells (Figures 2 and 3). Bcl-2, thus, seems not to be main player in ATRA + DAC + SAHA-induced apoptosis in HL-60 cells.

Attenuation of survivin expression induced by DAC + SAHA treatment persisted in HL-60 cells treated by DAC + SAHA + ATRA combination (Figure 3). Delocalization of centromere-related survivin was lower in comparison with DAC + SAHA treatment, but the fraction of mitotic cells significantly decreased although the proportion of cells in G2/M phase was stable. We conclude that ATRA potentiates DAC + SAHA-induced apoptosis preferentially in mitotic cells with delocalized survivin. This fact may be also one of the reasons that CML-T1 cells, where survivin is localized correctly after DAC + SAHA treatment, are resistant to ATRA addition to epigenetic drugs combination.

However, survivin repression is often regulated by p53-dependent pathway [8, 39] or contingent on presence of cyclin dependent kinase inhibitor p21 [37, 40]. Proteins p53 and p21 seem to collaborate also in ATRA-induced p21 enhancement. Much evidence of p21 induction after ATRA treatment (3–10 *μ*M) is possible to find for cells disposing of functional p53 [25, 41–43], enhancement of SAHA-induced p21 increase by ATRA was also described for wtp53 SH-SY5Y cells [44]. On the other hand, induction of apoptosis without p21 enhancement was documented for lung cancer cells [45]. We detected p21 induction together with p53 and Puma increase in CML-T1 cells after both DAC + SAHA and DAC + SAHA + ATRA treatment, enhanced p21 expression was detected also in ATRA-treated CML-T1 (Figure 6). By contrast, p21 protein was hardly detectable in ATRA-treated HL-60 cells and diminution of DAC + SAHA-induced p21 expression was observed after DAC + SAHA + ATRA treatment (Figure 3). Taking together, p53-dependent mechanism of p21 induction by both DAC and ATRA and p53-independent p21 enhancement induced by SAHA are concerned in final p21 level in tested cells.

Induction of reactive oxygen species after ATRA treatment is widely documented [24, 43, 46, 47]. In neuroblastoma cells, ROS generation induced by 5–10 *μ*M ATRA caused lipid peroxidation [46] and interplay between catalase, and ATRA was found during macrophage differentiation [48]. Recently, Wang et al. [26] detected ROS in HL-60 cells, but significant ROS induction was documented as late as after 5 days of ATRA treatment. In our experiments, ROS generation has been observed already 48 h after 1 *μ*M ATRA addition to HL-60 cells, but due to H_2_DCFDA intensity peak broadening statistical significance of mean intensity position shift was low (*P* = 0.062). However, addition of ATRA to HL-60 cells concurrently with DAC + SAHA led to statistically significant (*P* < 0.01) augmentation of ROS generation in comparison with DAC + SAHA-induced radicals (Figure 4(a)). Free radical scavenger N-acetyl-cysteine (NAC) lowered DAC + SAHA + ATRA-induced ROS generation as well as extent of apoptosis assessed by Annexin V staining and subG1-phase fraction of cells in cell cycle analysis. Similar relationship between ROS presence and the extent of apoptosis was documented using pan-caspase inhibitors z-VAD-fmk or Q-VD-OPh (Figure 4(b)). Q-VD-OPh is known to inhibit caspase activity of HL-60 cells already at 2 *μ*M, while higher concentration of Q-VD-OPh was reported to prevent PARP fragmentation or loss of cellular adhesiveness [49]. In our experiments, 30 *μ*M Q-VD-OPh completely cancelled DAC + SAHA + ATRA-induced apoptosis and simultaneously almost fully abolished ROS generation. Both the NAC and the Q-VD-OPh prevented PARP fragmentation and reverted caspase-3/7 activation as measured with fluorogenic substrate cleavage (data not shown). Therefore, significant role of oxidative stress in DAC + SAHA + ATRA-induced apoptosis is evident. ROS generation together with survivin deregulation has already been documented in several studies. ROS-dependent survivin downregulation induced by sulindac and SAHA combination was detected in human lung cancer cells [47]. Downregulation of survivin by indomethacin sensitized ROS-dependent apoptosis in TRAIL-resistant melanoma cells [50]. ROS production was observed in dentatin-induced prostate cancer cells apoptosis accompanied by Bcl-2 and survivin downregulation [51]. In neuronal tumor cells, high expression of mitochondrial survivin inhibited BAX relocalization into mitochondria and subsequent release of cytochrome c and ROS production [52]. Therefore, SAHA-induced survivin deregulation together with DAC or DAC + ATRA-induced ROS generation could also be the mechanism of effect of DAC + SAHA + ATRA action on HL-60 cells.

Expression of proapototic BAX protein only moderately increased in response to DAC + SAHA + ATRA treatment of HL-60 cells, but its mitochondrial localization in apoptotic cells indicating mitochondrial apoptotic pathway was found like it was documented in DAC + SAHA treated cells [5]. Negative correlation between survivin and BAX expression was detected in DAC + SAHA + ATRA-treated cells by flow-cytometry confirming again the relationship between loss of survivin expression and apoptosis (Figure 5).

Finally, no effect of studied combination of drugs on peripheral blood lymphocytes designates this combination to possible therapeutic utilization.

## 5. Conclusion

ROS generation and survivin downregulation were identified as main players in DAC + SAHA-induced apoptosis in p53-deficient leukemic cell line HL-60. Significant downregulation of both mRNA and protein expression of overall survivin together with delocalization of centromere-related survivin in mitotic cells was detected. We conclude that dysfunction of cell cycle regulating role of survivin contributes to apoptosis in HL-60 cells.

ATRA addition to DAC + SAHA combination further enhances apoptosis derived by survivin downregulation and ROS generation in p53-deficient cells. Both apoptosis and ROS generation were significantly reduced by radical scavenger N-acetyl-cysteine as well as by caspase inhibitors. No intensification of DAC + SAHA-induced effect by ATRA was observed in p53wt cell line CML-T1, where p53-dependent apoptosis was not accompanied with ROS generation and survivin decrease. Apoptotic process was not induced by DAC + SAHA + ATRA treatment of peripheral blood lymphocytes of healthy donors. This suggests possible usefulness of the combination of low concentrated DAC, SAHA, and ATRA in therapy of tumors with p53 dysfunction.
